# Therapeutic Opportunities in Melanoma Through PRAME Expression

**DOI:** 10.3390/biomedicines13081988

**Published:** 2025-08-15

**Authors:** Mislav Mokos, Ivana Prkačin, Klara Gaćina, Ana Brkić, Nives Pondeljak, Mirna Šitum

**Affiliations:** 1 Department of Dermatology and Venereology, Sestre Milosrdnice University Hospital Center, 10000 Zagreb, Croatia; mislavmokos50@gmail.com (M.M.); ivana.ljubicic.dr@gmail.com (I.P.); klaragacina@gmail.com (K.G.); brkic.ana1311@gmail.com (A.B.); mirna.situm@kbcsm.hr (M.Š.); 2 School of Medicine, University of Split, 21000 Split, Croatia; 3 Dermatovenereology Department, General Hospital Sisak, 44000 Sisak, Croatia; 4 School of Dental Medicine, University of Zagreb, 10000 Zagreb, Croatia; 5 Croatian Academy of Sciences and Arts, 10000 Zagreb, Croatia

**Keywords:** PRAME, melanoma, immunohistochemistry, cancer-testis antigens, immunotherapy

## Abstract

**Background:** Melanoma is one of the most aggressive types of skin cancer. Its diagnosis appears to be challenging due to morphological similarities to benign melanocytic lesions. Even though histopathological evaluation is the diagnostic gold standard, immunohistochemistry (IHC) proves to be useful in challenging cases. Preferentially Expressed Antigen in Melanoma (PRAME) has emerged as a promising diagnostic, prognostic, and therapeutic marker in melanoma. **Methods:** This review critically examines the role of PRAME across clinical domains. It presents an evaluation of PRAME’s diagnostic utility in differentiating melanomas from benign nevi, its prognostic significance across melanoma subtypes, and therapeutic applications in emerging immunotherapy strategies. An extensive analysis of the current literature was conducted, with a focus on PRAME expression patterns in melanocytic lesions and various malignancies, along with its integration into IHC protocols and investigational therapies. **Results:** PRAME demonstrates high specificity and sensitivity in distinguishing melanoma from benign melanocytic proliferations, particularly in challenging subtypes such as acral, mucosal, and spitzoid lesions. Its overexpression correlates with poor prognosis in numerous malignancies. Therapeutically, PRAME’s HLA class I presentation enables T-cell-based targeting. Early-phase trials show promising results using PRAME-directed TCR therapies and bispecific ImmTAC agents. However, immune evasion mechanisms (i.e., heterogeneous antigen expression, immune suppression in the tumor microenvironment, and HLA downregulation) pose significant challenges to therapy. **Conclusions:** PRAME is a valuable biomarker for melanoma diagnosis and a promising target for immunotherapy. Its selective expression in malignancies supports its clinical utility in diagnostic precision, prognostic assessment, and precision oncology. Ongoing research aimed at overcoming immunological barriers will be essential for optimizing PRAME-directed therapies and establishing their place in the personalized management of melanoma.

## 1. Introduction

Melanoma is one of the deadliest types of skin cancer, and it remains a growing public health concern worldwide. Epidemiological assessments show that, on the global scale, there were 325,000 new cases of melanoma in 2020, and 57,000 melanoma patients died during that year [1]. Furthermore, newer epidemiological data from the US estimate that the incidence of melanoma of the skin will reach 104,960 in 2025 in the US alone, with an estimated 8430 deaths [2].

Although early-stage melanoma can often be successfully treated with surgical removal, outcomes for advanced cases are still generally unfavorable. Early and precise identification of melanoma is crucial for improving survival rates. However, its resemblance to benign pigmented skin lesions frequently complicates the diagnostic process [3].

Diagnosis commonly relies on the histopathological evaluation of tissue samples, a method that is typically reliable [4]. Nevertheless, distinguishing melanoma from benign melanocytic lesions is sometimes challenging, even for experienced pathologists. This challenge is amplified when dealing with tumors that exhibit similar histopathologic features (i.e., atypical nevi or rare melanoma subtypes like amelanotic and desmoplastic melanoma). In such diagnostically ambiguous cases, immunohistochemistry (IHC) is often used to reach the definitive diagnosis [5].

One important protein, Preferentially Expressed Antigen in Melanoma (PRAME), has recently drawn a lot of interest due to its selective expression in malignant cells. PRAME’s tumor-specific expression makes it a promising biomarker, opening up promising possibilities across multiple clinical domains, from diagnosis to prognosis and therapy [6].

From the therapeutic perspective, PRAME and other tumor-associated antigens (TAAs) present favorable targets for immunotherapy. These proteins, found predominantly, or in much higher quantities, in tumor cells compared to normal tissue, allow for precision-based interventions. By directing the immune system against these antigens, particularly by using T cells or vaccine-based approaches, it becomes possible to maximize tumor eradication while minimizing harm to healthy cells [6].

This article presents a narrative review of the literature on PRAME in melanoma. Relevant publications were identified through comprehensive searches of major biomedical databases (PubMed and Scopus). This review explores PRAME’s multifaceted role in everyday clinical practice for managing melanoma. It covers PRAME’s diagnostic and therapeutic value, as well as its potential as a prognostic biomarker that could influence patient monitoring and treatment decisions.

## 2. Background

### 2.1. Tumor-Associated Antigens (TAAs)

TAAs are a diverse group of proteins that are significantly more expressed in malignant cells compared to benign cells [7]. This makes them potential biomarkers for cancer diagnosis, prognosis, and therapeutic intervention. Examples of well-known TAAs include alpha-fetoprotein (AFP), carcinoembryonic antigen (CEA), prostate-specific antigen (PSA), and several heat shock proteins (HSPs), all of which have demonstrated clinical relevance across a spectrum of malignancies such as liver, colorectal, gastric, prostate, and lung cancers [8,9]. TAAs are commonly present in blood serum, which is beneficial for non-invasive monitoring of disease onset, progression, and response to treatment [9].

Furthermore, TAAs have emerged as interesting therapeutic targets. Since they are often involved in key processes like tumor cell proliferation, immune evasion, and metastasis, TAAs are being explored in the development of immunotherapies (including cancer vaccines, monoclonal antibodies, and radioimmunoconjugates) that aim to elicit or enhance the immune system’s ability to recognize and destroy cancer cells [10,11,12]. A well-known subclass of TAAs, cancer-testis antigens (CTAs), are particularly attractive because they are expressed only in immune-privileged germline tissues and a wide array of tumors [13].

However, the clinical application of TAAs still faces many challenges. Numerous TAAs lack sufficient specificity or sensitivity when used as diagnostic or prognostic markers, which can lead to false positives or negatives in cancer screening and monitoring [14]. Thus, in order to improve accuracy, diagnostic panels with multiple TAAs are being developed [15].

### 2.2. Cancer-Testis Antigens (CTAs)

The discovery of CTAs has significantly advanced our understanding of tumor immunology and opened new pathways for developing targeted therapies [13]. CTAs represent a distinct group of TAAs that are expressed in various cancers but are otherwise limited to germline tissues such as the testes, placenta, and ovaries in healthy adults [16]. Over time, newer techniques such as SEREX (serological analysis of cDNA expression libraries) have replaced older methods, leading to a dramatic increase in the number of known CTAs, this group now numbering over 200 individual proteins [17].

One of the defining features of CTAs is their expression pattern. Though originally thought to be restricted to germline tissues, recent findings indicate that some CTAs, including PRAME, may also be expressed in adrenal and endometrial tissues [13,17]. CTAs are generally grouped into two categories based on their chromosomal location: one group encoded on the X chromosome, and the other on autosomes or the Y chromosome [18]. On the other hand, the role of CTAs in the differentiation and proliferation of germ cells is still poorly understood.

The reason why CTAs present an appealing new possibility in cancer therapy is likely due to their immunogenic nature and selective expression in malignant cells. This makes them ideal targets for innovative immune-based treatments, such as T-cell therapies and cancer vaccines [19]. For these immunotherapies to be effective, identifying antigens that are presented on cancer cells’ HLA class I molecules but absent from normal somatic cells is crucial. The unique properties of CTAs position them at the forefront of efforts to design next-generation, precision-based cancer immunotherapies [20].

### 2.3. Preferentially Expressed Antigen in Melanoma (PRAME)

Initially discovered during research on melanoma cell lines, PRAME has since garnered attention as a promising immunotherapeutic target [21]. It belongs to the family of CTAs and is aberrantly expressed in a wide range of solid tumors and hematologic malignancies [22]. What makes PRAME particularly significant in cancer immunology is its presentation on human leukocyte antigen class I (HLA-I) molecules, enabling T-lymphocytes to recognize and potentially eliminate cancer cells [23]. Structurally, PRAME is a 509-amino-acid protein that resides in both the nucleus and cytoplasm of various human cell types [24]. Its gene is located on chromosome 22 (specifically at 22q11.22), surrounded by a cluster of immunoglobulin-related genes. This combination of tissue-restricted expression, tumor-specific upregulation, and immunogenicity highlights PRAME’s potential use in immunotherapy [25].

Recent investigations into the molecular mechanisms driving tumorigenesis have brought attention to PRAME. Originally restricted to germline tissues, PRAME’s aberrant activation in somatic cells contributes to cancer development through multiple pathways, including suppression of retinoic acid (RA) signaling, induction of epithelial-to-mesenchymal transition (EMT), and modulation of the tumor immune microenvironment [26].

The interaction between PRAME and the RA signaling pathway (a regulatory system for cellular differentiation, growth inhibition, and programmed cell death) has a central role in the biology of a malignant cell [23]. In healthy cells, RA, a derivative of vitamin A, binds to nuclear receptors (RARα, RARβ, or RARγ). This activates the transcription of genes that promote cell cycle arrest and differentiation. Therefore, this pathway has strong anti-proliferative effects, and it is fundamental in various therapies like all-trans RA (ATRA) treatment in acute promyelocytic leukemia [27]. However, PRAME can inhibit this protective mechanism by binding to RA receptors, which suppresses downstream gene activation [28]. This competitive inhibition may explain why PRAME is frequently overexpressed in highly proliferative malignant cells, which are commonly resistant to growth suppression and apoptosis.

Also, PRAME contributes to cancer progression by promoting EMT. This is a process where epithelial cells acquire mesenchymal traits, losing adhesion and gaining enhanced motility [29]. This transition enables tumor cells to migrate, invade, and establish metastatic lesions. Studies have shown that PRAME, along with other CTAs such as CT45A1 and MAGEC2, facilitates EMT, potentially through pathways like β-catenin signaling. However, the exact mechanism remains under investigation [30]. Namely, metastatic tumors often show elevated CTA expression compared to their primary counterparts, which additionally underscores the association between CTAs and the development of metastases [31].

Beyond influencing tumor cell behavior directly, PRAME also plays a significant role in shaping the immune landscape of cancer. Tumors with high PRAME expression tend to have “cold” immune profiles. This means that their antigens are less presented on cell surface and their immune checkpoint molecules are highly expressed, which is useful for evading T-cell recognition and cytolysis [32]. In a breast cancer model, silencing PRAME in MDA-MB-468 cells was shown to enhance T-cell-mediated cytotoxicity and decrease the expression of immune checkpoint regulators [33]. In contrast, PRAME overexpression was found to reduce immune cell activation [34].

## 3. PRAME Expression and Its Clinical Implications

Although PRAME is not uniformly expressed across all cancers, it is frequently overexpressed in a wide array of malignancies (Table 1). Initially identified in cutaneous melanoma, where it was first characterized, PRAME has since been detected in numerous other tumor types, such as leukemia, neuroblastoma, non-small-cell lung cancer (NSCLC), breast cancer, ovarian cancer, and several sarcomas [22,35,36]. Its expression patterns across these various malignancies can differ significantly. Therefore, this variability affects disease prognosis and treatment strategies [37,38,39,40].

Recent studies have highlighted the clinical significance of PRAME expression in acute myeloid leukemia (AML). Namely, PRAME was proven to be both a CTA and a promising target for immunotherapy. This underscores its utility in monitoring minimal residual disease (MRD) and predicting relapse, particularly in patients without clear genetic markers [40,41]. Furthermore, it was found that PRAME mRNA levels are significantly elevated in AML patients and associated with poor prognosis [42]. Additionally, research comparing PRAME and WT1 expression patterns showed that rising PRAME levels during follow-up were predictive of clinical relapse, further underscoring its potential as a dynamic biomarker for MRD surveillance [43]. Similarly, in melanoma, high PRAME expression is often associated with more aggressive tumor behavior and a poorer clinical outcome [44]. By contrast, the role of PRAME in breast and ovarian cancers is less well defined, though emerging evidence suggests it may correlate with adverse prognosis in these settings as well [45,46,47,48].

PRAME expression is notably prevalent in neuroblastoma, particularly in the disease’s advanced stages. The rate of PRAME expression was reported to be up to 93% in primary neuroblastoma samples and 100% in patients with advanced disease. This expression was significantly associated with a more advanced tumor stage and an older age at diagnosis [49]. Furthermore, elevated PRAME levels have been linked to poorer overall survival (OS) and event-free survival (EFS) in neuroblastoma patients. Higher PRAME expression, as assessed through digital image analysis, has been associated with worse prognosis according to recent findings [50].

In addition, high expression levels have been observed in myxoid liposarcomas, synovial sarcomas, and uterine carcinosarcomas. In myxoid liposarcomas, PRAME expression was detected in 90% of cases, correlating with higher tumor grade and advanced clinical stage [51]. Also, elevated PRAME expression in sarcomas has been associated with adverse clinical features, including larger tumor size, the presence of necrosis, and higher histological grade. These factors contribute to a poorer prognosis, suggesting that PRAME could serve as a prognostic biomarker in certain sarcoma subtypes [51].

Moreover, elevated PRAME expression has been noted in NSCLC, with varying expression rates across different histological subtypes [52]. Namely, a study found that PRAME was expressed in 59.2% of patients with squamous cell carcinoma and 32.2% of patients with adenocarcinoma of the lungs [53]. This expression pattern suggests a higher prevalence in squamous cell carcinoma compared to adenocarcinoma. However, PRAME’s potential role as a prognostic marker remains unclear, with studies indicating no significant association between PRAME expression and OS [53].

Furthermore, the expression of PRAME in non-melanoma skin cancers (NMSCs) remains underexplored. Studies to date present a mixed picture: some report widespread and intense staining in basal cell carcinomas and, less frequently, in Merkel cell carcinomas (MCCs), while others show minimal or no expression in most NMSCs, with the exception of certain MCCs exhibiting high-intensity expression [36,54,55].

**Table 1:** PRAME expression in numerous neoplasms and its clinical relevance.

Neoplasm Type	Cohort Size	Expression Rate	Relevance	Reference
AML	>2000 AML patients (children and young adults)	30% (pediatric leukemias)	- The practicality and therapeutic impact of using next-generation PRAME-specific mTCRCAR T cells	[41]
	50 (40 AL patients and 10 healthy individuals)	PRAME mRNA: 80% in AL and 20% in controls	- Potentially useful as a marker for tracking MRD - Its presence has been associated with unfavorable outcomes in AML patients	[42]
	204 AML patients, 22 healthy controls	4.01% (AML patients)	- Increasing PRAME levels over time predicted clinical relapses, highlighting its value as a dynamic MRD monitoring biomarker	[43]
Breast cancer	220 cases	24.1%	- PRAME is more frequently expressed in HER2+ and triple-negative breast cancers and may serve as an immunotherapy target - However, it is not an independent prognostic factor	[45]
Ovarian cancer	119 cases of EOC, 17 healthy ovaries	~60% of primary EOCs	- PRAME is often expressed in EOC and HGSC, supporting its potential as an immunotherapy target, possibly enhanced by epigenetic agents like decitabine	[46]
Neuroblastoma	94 primary neuroblastoma patients	93% in primary neuroblastoma (and 100% in advanced disease)	- PRAME expression significantly influences neuroblastoma outcomes, making it a promising target for immunotherapy	[49]
Sarcoma	93 myxoid liposarcoma samples, 46 dedifferentiated liposarcoma samples, 32 well-differentiated liposarcoma samples, and 14 pleomorphic liposarcomas samples	90% in myxoid liposarcomas, 43% in dedifferentiated liposarcomas, 9% in well-differentiated liposarcomas, and 50% in pleomorphic liposarcomas	- High PRAME levels in sarcomas are linked to aggressive features like larger tumors, necrosis, and higher grade - These associations point to PRAME’s potential role as a prognostic biomarker in specific sarcoma subtypes	[51]
NSCLC	377 specimens	49.9%	- PRAME expression in NSCLC correlates with factors like smoking status and tumor histology - Its prognostic value is uncertain, as no clear link to OS has been found	[53]
NMSCs	42 BCCs	62%	- PRAME’s limited specificity as an IHC marker for NMSCs reduces its utility in diagnostic surgical pathology	[36]
	27 MCCs	30%	- IHC offers a reliable and cost-effective way to identify PRAME-positive cancers for potential immunotherapy	[36]
Melanoma	155 primary melanomas	83.2%	- PRAME IHC may aid in confirming a suspected melanoma diagnosis - It could also help assess surgical margins in known PRAME-positive melanomas - PRAME expression in benign skin lesions poses diagnostic challenges, requiring further study	[22]
Metastatic melanoma	100 metastatic melanomas	92%	- Widespread PRAME expression in metastatic melanoma supports its potential as an immunotherapy target	[22]
Mucosal melanoma	29 mucosal melanoma cases	83.3%	- High PRAME expression in mucosal melanomas suggests it could be a promising target for future therapies	[56]
Acral melanoma	10 acral melanomas	100% in acral melanoma	- Strong PRAME expression is a highly sensitive and specific diagnostic marker for acral melanomas, outperforming p16 IHC	[57]
Ocular melanoma	389 UM patients	84–100%	- PRAME independently predicts metastasis risk in uveal melanoma, improving prognostic testing and precision care	[58]
	40 (30 invasive conjunctival melanoma samples and 10 in situ conjunctival melanoma samples)	57% in invasive melanoma samples and 70% in situ melanoma samples	- Diffuse 4+ PRAME staining is highly specific for malignant conjunctival melanocytic lesions, aiding in melanoma diagnosis	[59]

Abbreviations: AL (acute leukemia), AML (acute myeloid leukemia), BCC (basal cell carcinoma), EOC (epithelial ovarian cancer), HER2 (human epidermal growth factor receptor 2), HGSC (high-grade serous carcinoma), IHC (immunohistochemistry), MCC (Merkel cell carcinoma), MRD (minimal residual disease), NMSC (non-melanoma skin cancer), NSCLC (non-small cell lung cancer), OS (overall survival), PRAME (Preferentially Expressed Antigen in Melanoma), UM (uveal melanoma).

## 4. PRAME as a Diagnostic and Prognostic Biomarker in Melanocytic Tumors

The most recent WHO classification of skin tumors segregates melanocytic skin tumors into melanocytic neoplasms in intermittently sun-exposed skin and melanocytic neoplasms in chronically sun-exposed skin. The first category is further differentiated into nevi, melanocytomas, and melanoma in intermittently sun-exposed skin. Moreover, the second category contains only one subcategory, and that is melanoma in chronically sun-exposed skin [60]. Despite advances in immunohistochemistry, distinguishing melanoma from benign melanocytic proliferations remains a diagnostic challenge, particularly in histologically ambiguous cases. Recent studies have highlighted the limitations of traditional markers in certain contexts, underscoring the need for more reliable biomarkers (Table 2). PRAME has emerged as a promising candidate to help address this gap, since it is increasingly recognized as a useful IHC marker in the diagnosis and prognosis of melanocytic tumors [22]. Its utility lies in its distinct expression profile: it is typically absent or weak in benign nevi but strongly expressed in melanomas [22]. This has made it a highly sensitive (>90%) and specific tool for distinguishing malignant melanomas from benign melanocytic lesions, including in lymph nodes, where it helps differentiate metastatic disease from nodal nevi [22,61]. However, PRAME expression alone is insufficient for definitive diagnosis, especially in metastases [22,61]. Importantly, PRAME may also carry prognostic value and offers promise as a therapeutic target in melanoma immunotherapy [62,63,64].

In mucosal melanomas, rarer than their cutaneous counterparts, PRAME is often expressed at high levels and correlates with poorer outcomes. While it was found that benign mucosal lesions in the head and neck mostly lack PRAME staining, Toyama et al. showed a link between strong PRAME expression and unfavorable prognosis in mucosal melanomas [56,80]. Notably, this expression appears independent of NRAS mutation status, even though both are individually linked to poor prognosis [56]. A 60% threshold of PRAME-positive cells was proposed as an effective diagnostic cutoff for distinguishing benign from malignant mucosal melanocytic lesions [22].

Acral melanomas present another diagnostic challenge due to histologic overlap with acral dysplastic and Spitz nevi, which may display atypical features [57,81,82]. However, studies show that PRAME is consistently absent in benign acral nevi but universally 4+ positive in acral melanomas, regardless of Breslow thickness [57,82]. In lesions with lower staining intensity (1+ to 3+), further testing is often needed based on clinical context. Overall, PRAME outperforms p16 IHC in these scenarios, supporting its role in difficult diagnostic cases [57].

On the other hand, many challenges arise from the fact that PRAME has highly variable expression in different tumor types [81,83,84]. For instance, some subungual melanomas and acral melanomas can have a low PRAME expression, while certain benign nevi may test positive [85]. Moreover, evidence for PRAME’s use in amelanotic melanoma remains inconclusive, which shows the need for further studies on this topic [86].

Furthermore, in subungual melanocytic lesions, PRAME has shown great diagnostic potential. Namely, it successfully distinguishes benign subungual melanocytic proliferation from malignant melanoma [87,88]. It also aids in detecting melanoma in small or subtle samples, though limitations remain in cases with minimal morphological clues [89].

PRAME has also emerged as a key biomarker in ocular melanomas. In uveal melanoma, which originates in the uvea and differs genetically from cutaneous melanoma, PRAME overexpression is linked to poor prognosis and increased liver metastasis risk [28,58]. Conjunctival melanoma, though less common, shares histopathologic traits with other melanoma subtypes, and PRAME is under investigation as a diagnostic and prognostic marker here as well [59,90].

Taken together, these findings highlight that the prognostic significance of PRAME may vary depending on tumor context and subtype. This variability emphasizes the need for further research and careful interpretation when considering PRAME as a prognostic biomarker in clinical decision-making.

## 5. Advancing Melanoma Diagnosis with PRAME Immunohistochemistry

Melanoma remains a challenging clinical and histopathologic diagnosis due to its morphological overlap with benign melanocytic proliferations, such as common nevi or dysplastic nevi [91,92]. This potentially problematic diagnostic procedure can even result in delayed or inappropriate treatment, which highlights the importance of reliable diagnostic tools [93].

Clinically, the initial clinical assessment of melanocytic lesions often involves the ABCDE criteria (asymmetry, border irregularity, color variation, diameter exceeding six millimeters, and evolution over time) [94]. Even though these criteria are useful during the basic clinical assessment, they are not definitive (79,80). In cases where the clinical and dermoscopic diagnosis remains uncertain, tissue biopsy is necessary [95,96]. Pathologists then evaluate architectural and cytologic features, including architectural disorder, cytologic atypia, increased mitotic activity, and failure of maturation with progressive descent into the dermis [97].

Despite these standard diagnostic protocols, certain lesions remain difficult to classify. In such instances, IHC (Table 2) and cytogenetic analyses are employed as adjunctive diagnostic modalities [4,98,99]. Common IHC markers used in melanoma diagnosis include S100, SOX10, Melan-A, and HMB-45 [65,66,67]. S100 and SOX10 are highly sensitive for melanocytic differentiation but lack specificity, as they stain both benign and malignant melanocytes [4,68,69]. Melan-A is similarly limited by its inability to differentiate malignant from benign lesions [70]. HMB-45 offers slightly improved specificity but remains insufficient in certain histologic contexts [67,71]. To overcome these limitations, ancillary cytogenetic tests such as fluorescence in situ hybridization (FISH) and single nucleotide polymorphism (SNP) arrays are used to detect genetic alterations associated with malignancy [100,101].

PD-L1 immunohistochemistry (IHC) has been extensively studied as a prognostic and predictive biomarker in melanoma immunotherapy, though results have been variable. While some studies have shown associations between PD-L1 expression and clinical outcomes, including overall survival and response to immune checkpoint inhibitors [72,73], others have demonstrated that patients lacking PD-L1 expression may still derive significant clinical benefit from PD-1 blockade [74]. Moreover, the prognostic value of PD-L1 often depends on the tumor microenvironment context, including the presence of CD8+ tumor-infiltrating lymphocytes and other immune parameters [72,75]. This inconsistency highlights the limitations of PD-L1 as a standalone biomarker and underscores the need for alternative or complementary targets, such as PRAME, for stratifying melanoma patients and guiding immunotherapeutic strategies.

PRAME has recently emerged as a promising IHC marker due to its restricted expression in benign tissues and frequent overexpression in malignant melanocytic lesions. Numerous studies have proven the diagnostic value of PRAME, which is being increasingly adopted in routine clinical practice [22,76,77,78]. In one study, PRAME was found to be diffusely positive in 92% of metastatic melanomas and in 92% of primary cutaneous melanomas, excluding desmoplastic variants [22]. Importantly, among 140 benign cutaneous and nodal nevi, 86.4% were PRAME-negative, suggesting high specificity [22]. A separate investigation using immunocytochemical methods reported PRAME positivity in 85.4% of cutaneous melanoma metastases [79]. While these sensitivity rates are somewhat lower than those observed for traditional melanocytic markers such as S100, SOX10, and Melan-A, PRAME distinguishes itself by offering significantly higher specificity, thereby reducing the risk of overdiagnosis [66,79].

The expression of PRAME varies across melanoma subtypes [22,102,103,104]. Desmoplastic melanoma, a neurotropic and often diagnostically challenging subtype, expresses PRAME in only approximately 35% of cases, limiting its utility in this context [22]. In contrast, acral melanomas are often histologically indistinguishable from benign acral nevi and exhibit high rates of PRAME positivity [81]. A recent study demonstrated that 87.1% of acral melanomas were PRAME-positive, whereas 82.5% of benign acral lesions were either faintly positive or negative [81]. In a separate cohort, all examined cases of acral melanoma tested positive for PRAME, while none of the benign, dysplastic, or Spitz-type acral nevi showed PRAME expression [57].

Additional research has explored the concordance between PRAME IHC and cytogenetic analyses [86,90,105,106,107,108]. In one study involving 110 diagnostically challenging melanocytic lesions, PRAME immunostaining demonstrated 90% concordance with cytogenetic findings and 92.7% concordance with final clinical-pathologic diagnoses [108]. These results suggest that PRAME IHC may serve as a reliable alternative or adjunct to molecular testing in routine practice.

Despite these associations, the prognostic implications of PRAME remain a subject of debate [109,110,111]. Some studies have found no significant differences in PRAME expression between primary and metastatic cutaneous melanomas, possibly due to consistently high expression levels in both stages of the disease [109]. These findings underscore the need for further research into the prognostic value of PRAME across different stages and subtypes of melanoma.

Finally, PRAME has shown utility in the detection of microsatellites in melanoma, which are often missed using traditional H&E staining [89]. Since PRAME expression is retained in nearly all melanoma cells within PRAME-positive tumors, even small satellite deposits can be identified more easily, potentially improving staging accuracy [89].

## 6. PRAME in Spitzoid Melanocytic Lesions

The role of PRAME IHC remains a subject of debate because PRAME is inconsistently expressed in various benign and malignant entities [86,112]. Even though PRAME was originally associated with malignancies, it has also been detected in benign lesions such as solar lentigines, normal skin, and notably, Spitz nevi [83,86,113]. This problematic aspect of PRAME’s role as a diagnostic marker is particularly highlighted in studies that have evaluated its diagnostic utility in differentiating spitzoid lesions [83,105,113,114,115].

A recent study revealed that strong, diffuse PRAME positivity was present in 20% of Spitz nevi, absent in all atypical Spitz tumors, and prominent in 82% of spitzoid melanomas [83]. This suggests that PRAME could be useful in distinguishing spitzoid melanoma from benign Spitz nevi [83]. The conclusion of this study aligns with earlier observations, which reported that most Spitz nevi showed no PRAME expression, only one out of eleven cases displayed diffuse positivity, and two had focal reactivity. Bearing this in mind, the authors proposed that widespread PRAME expression is more suggestive of malignant behavior in melanocytic lesions [112].

In contrast to earlier findings, some researchers have emphasized the need for caution when interpreting PRAME staining results [113]. Namely, it was found that most Spitz nevi and atypical Spitz tumors completely lacked PRAME expression. However, exceptions existed, since isolated cases showed diffuse positivity [113]. These outliers underscore the limitations of relying solely on PRAME for diagnostic precision.

Further complicating the interpretation of PRAME, one study investigated the correlation between PRAME immunostaining and FISH outcomes in diagnostically ambiguous melanocytic lesions [105]. There was minimal association between these two diagnostic methods, which raises concerns about the reliability of PRAME as an independent diagnostic marker in spitzoid tumors [105]. The study also brought attention to the lack of consensus regarding the percentage of PRAME-positive melanocytes required to classify a lesion as “diffusely positive” [105]. This lack of standardization could be the reason behind significant interpretive variability across studies.

In another study, PRAME expression was clearly linked to the diagnosis of atypical spitzoid melanocytic proliferations [114]. This finding clearly supports the potential diagnostic value of PRAME. However, the importance of a cautious and integrative approach was highlighted in this study, since the risk of both false-positive and false-negative interpretations was noted [114].

A more recent study analyzed a series of spitzoid lesions, reporting diffuse PRAME positivity in approximately 27% of cases [115]. Of those, seven lesions were both FISH-positive and diagnosed as spitzoid melanomas [115]. The remaining eight PRAME-positive but FISH-negative cases included seven atypical Spitz tumors and one spitzoid melanoma, reflecting a degree of discordance [115]. This study did not demonstrate a significant association between PRAME expression and FISH results, which reinforces concerns about the marker’s limitations [115].

Taken together, these findings illustrate the diagnostic ambiguity that can arise from variable PRAME expression in spitzoid lesions. While diffuse PRAME positivity may suggest malignant potential, its occasional presence in benign Spitz nevi underscores the need for a multimodal diagnostic approach. Adjunctive techniques, such as FISH, p16 immunostaining, or molecular profiling, should be considered in diagnostically challenging cases to enhance accuracy and minimize misclassification.

## 7. Immunotherapy and the Emerging Role of PRAME

One of the biggest challenges in oncology is the difficulty of distinguishing cancer cells from healthy somatic cells. This challenge arises from their striking genetic and structural similarities, since malignant cells often appear nearly identical to their normal counterparts [116]. However, cancer cells diverge through upregulated genetic programs that enable hallmark traits such as immune evasion, sustained proliferation, resistance to apoptosis, chronic inflammation, and replicative immortality [117].

Traditional treatments like surgery, chemotherapy, and radiation have significantly improved survival rates over the years. On the other hand, these interventions come with major drawbacks. Namely, they often damage healthy tissues, and they are also limited in preventing recurrence and metastasis. The undiscriminating nature of these therapies has urged clinicians and researchers to focus on more selective and better-tolerated therapeutic options [118].

This need for specificity in treatment was the reason why the focus was shifted onto immunotherapy. This form of therapy relies on the immune system to selectively target tumor cells. Early immunotherapeutic agents, such as interferon-alpha 2 (IFN-α2) and interleukin-2 (IL-2), were among the first to be approved by the FDA, and they function by enhancing T-cell proliferation and activity [119]. This was followed by the approval of immune checkpoint inhibitors (ICIs). ICIs are antibodies that block inhibitory receptors like cytotoxic T-lymphocyte-associated protein 4 (CTLA-4) and programmed death-ligand 1 (PD-L1) [120]. These advances in immunotherapy have significantly improved outcomes for patients with advanced melanoma, largely through the targeting of immune checkpoints that regulate T cell responses. Blockade of CTLA-4 was the first to demonstrate the potential of this approach, while inhibition of programmed cell death protein 1 (PD-1), the receptor for PD-L1, proved to be more effective and less toxic [121]. As a result, PD-1 inhibitors such as nivolumab and pembrolizumab have become central to melanoma treatment. Although PD-L1 is often discussed in this context, it is the ligand for PD-1, and current standard therapies in melanoma primarily target PD-1 itself, not PD-L1 [122,123].

Real-world data further support the utility of PD-1-based immunotherapy in metastatic melanoma. In a cohort of 522 patients, only 8.2% showed an early response (within three months), while 19% responded later, 37% had stable disease, and 35.8% were non-responders [124]. Early responders had favorable survival outcomes, but their long-term survival was not superior to that of late responders, indicating that delayed responses to PD-1 blockade can be equally durable. Notably, tumor PD-L1 positivity and normal CRP levels were independently associated with early response [124].

Despite these advances, the response rate to PD-1/PD-L1 monotherapy remains limited in a significant proportion of patients due to the multifaceted nature of tumor immune evasion [125]. Combination strategies (including PD-1/PD-L1 inhibitors with chemotherapy, radiotherapy, angiogenesis inhibitors, or other immunomodulatory agents) have shown promise in enhancing antitumor efficacy by acting on multiple phases of the cancer–immunity cycle and remodeling the tumor microenvironment. Emerging approaches, such as bispecific antibodies and personalized immunotherapy regimens, offer additional potential to boost therapeutic outcomes and overcome resistance mechanisms in melanoma and other cancers [126].

Clinical studies and meta-analyses have shown that both PD-1 and PD-L1 inhibitors exhibit strong antitumor activity in melanoma, with particularly high response rates compared to other malignancies. However, PD-L1 expression, while a potential indicator of response, is not a reliable standalone biomarker [127]. Combination therapy, particularly PD-1 inhibitors in conjunction with CTLA-4 blockade, has demonstrated improved response rates, especially in patients who are less likely to benefit from monotherapy [125,126,128,129,130,131]. This benefit, however, is accompanied by a higher incidence of immune-related adverse events that require close monitoring and management [132].

A recent meta-analysis including three randomized controlled trials and two retrospective studies found that combination therapy with ipilimumab (CTLA-4 inhibitor) and nivolumab (PD-1 inhibitor) significantly increased both overall response (OR: 2.144) and complete response (OR: 2.117) rates compared to monotherapy [133]. Subgroup analyses revealed that the combination with ipilimumab contributed most significantly to improved outcomes (overall response OR: 5.440), although it also led to a higher frequency of treatment-related adverse events (OR: 4.044 compared to nivolumab alone), underscoring the trade-off between efficacy and toxicity [133].

Building on this foundation, the field has advanced toward highly personalized cellular therapies. These include CAR-T cell therapy, where a patient’s T cells are genetically modified to express synthetic receptors that target tumor antigens, and the ex vivo expansion of antigen-specific T cells to boost the immune response [134]. A critical step of these therapeutic procedures is the identification of tumor-exclusive antigens presented on HLA class I molecules. This is important because they are used to distinguish cancer cells from normal tissue [135].

Among the most promising candidates are CTAs, which are normally found in immune-privileged germline tissues. However, they are also aberrantly expressed in tumor cells [136]. PRAME is a CTA that has attracted significant attention for its selective expression on malignant cells and presentation on HLA-I molecules. This makes PRAME an appealing target for a variety of immunotherapeutic approaches, including cancer vaccines and T-cell-based therapies currently under investigation [6]. As research advances, PRAME could easily become the next focal point of precision oncology.

### 7.1. PRAME as a Target in the Development of T-Cell Immunotherapies

The scientific search for more precise and effective cancer therapies has led to groundbreaking progress in harnessing T cells to recognize and eliminate malignant cells. Among the most promising advancements in this domain are two major strategies of adoptive cell transfer (ACT): the expansion of tumor-infiltrating lymphocytes (TILs) and the engineering of T cells with synthetic or modified receptors, including both chimeric antigen receptors (CARs) and T-cell receptors (TCRs) [137].

TIL therapy operates on the principle that the immune system already mounts a response against tumors. Lymphocytes that naturally infiltrate tumors are isolated after tumor resection, expanded ex vivo using growth-promoting interleukins such as IL-2, IL-7, or IL-21, and then reinfused into the patient. These enriched T-cell populations are capable of recognizing TAAs with high specificity [138]. Since its introduction in 1988 for metastatic melanoma, TIL therapy has demonstrated meaningful clinical benefits [139]. A meta-analysis found that 41% of melanoma patients previously treated with chemotherapy or radiation exhibited objective responses to TIL therapy, an encouraging statistic for a patient population with limited options [140].

To further improve the outcomes of TIL therapy, researchers have turned their attention to T cells that specifically recognize CTAs such as PRAME. Using proteasome cleavage analyses, scientists identified four high-affinity HLA-A*02:01-restricted epitopes derived from PRAME [141]. TILs targeting these epitopes have been detected in 36% of melanoma patients and up to 70% of those with AML [142,143].

CAR T-cell therapy is also an interesting new therapeutic modality. This process involves reprogramming T cells to express synthetic receptors that can recognize tumor antigens independently of HLA presentation [144]. These CARs confer robust cytotoxic activity upon antigen engagement, allowing modified T cells to release granzymes and perforin to destroy the target cells [145]. Unlike native TCRs, CARs bind directly to surface antigens, which limits their utility to extracellular targets [146]. Since PRAME is an intracellular antigen, conventional CAR T-cell strategies are not ideal for targeting it directly.

This limitation is especially relevant for PRAME, which resides intracellularly and thus escapes detection by standard CAR T-cells [40]. To overcome this, researchers are focusing on TCR-engineered T-cell therapies that maintain the ability to recognize peptide antigens presented on HLA class I molecules [147]. This makes PRAME a viable target for TCR-based approaches, particularly in tumors where it is abundantly expressed [47].

Several investigational therapies are currently being evaluated for their ability to target PRAME through engineered TCRs (Table 3). A notable example is a clinical trial assessing the safety and antitumor activity of BPX-701, a TCR-engineered autologous T-cell product targeting PRAME in patients with metastatic uveal melanoma [148]. This therapy features a built-in safety switch controllable via the drug rimiducid [148]. Similarly, IMA203, a PRAME-targeted TCR therapy, has demonstrated promising results in a phase 1 trial [149]. The safety profile has been shown to be manageable, and the efficacy signals are encouraging. Namely, the overall response rate was 52.5% and there was a confirmed response in nearly 29% of patients with advanced, PRAME-positive solid tumors such as melanoma and sarcoma [149]. The therapy showed rapid engraftment, long-term T cell persistence, and enhanced outcomes in patients receiving higher doses or those with stronger PRAME expression. Therefore, this provides support for further clinical development [149].

### 7.2. New Cancer Vaccines Based on PRAME as a Tumor Antigen

The transition of “cancer vaccines” from preventive tools to therapeutic agents has revolutionized the field of oncology [154,155]. These vaccines now aim to trigger a potent immune response against tumor-specific antigens by introducing either synthetic peptides, DNA or RNA sequences, often paired with immunostimulatory adjuvants [156,157]. This concept mirrors viral vaccine mechanisms but is applied post-diagnosis to treat existing cancers. Among the available platforms, peptide vaccines, dendritic-cell-based vaccines, and mRNA-based formats are currently under the most scrutiny [158,159,160]. Because of their restricted expression in normal tissue and elevated presence in tumors, CTAs have emerged as ideal targets, with PRAME garnering increasing interest (Table 3).

Although CTA-targeted peptide vaccines such as MAGE-A and NY-ESO-1 have induced T-cell and antibody responses in early trials, their clinical effectiveness has often faltered in later-phase studies. For instance, two phase III trials involving MAGE-A3 vaccines in NSCLC and melanoma failed to improve disease-free survival across more than 3500 participants [161,162]. Furthermore, preclinical studies in mice demonstrated promising CD4+ and CD8+ responses, leading to phase I investigations in primates and humans. However, these trials recorded only antibody production and minimal T-cell activation, particularly lacking cytotoxic CD8+ engagement [163]. This shortfall has prevented progression to phase II and III trials.

The weak cytotoxic response may be due to the immunological tolerance developed against PRAME, particularly in melanoma patients. One solution involves re-sensitizing or retraining the immune system using polyvalent vaccine approaches that target multiple antigens. In one study, a dual-target vaccine addressing both PRAME and prostate-specific membrane antigen (PSMA) triggered CD8+ T-cell expansion in 15 of 24 prostate cancer patients, with several showing sustained disease stability over a six-month period (this included one out of 10 patients with metastatic melanoma). These results suggest that broader antigen targeting may circumvent immune escape and enhance the durability of response [150].

Parallel innovation has produced alternative therapeutic avenues such as Immune mobilizing monoclonal T-cell receptors Against Cancer (ImmTACs), including bispecific molecules like brenetafusp (IMC-F106C), which are designed to bridge intracellular targets like PRAME and cytotoxic T cells [151]. Brenetafusp integrates a soluble TCR that binds HLA-presented PRAME peptides and a CD3-targeting domain that redirects T cells to tumor sites [151]. Clinical evaluation of patients with heavily pre-treated cutaneous melanoma revealed encouraging results: a 58% disease control rate, a median progression-free survival of 4.2 months, and manageable adverse effects such as mild cytokine release syndrome and rash. Additionally, 42% of PRAME-positive patients showed molecular responses through circulating tumor DNA analysis, indicating biologic activity of the treatment [151]. These findings supported further investigation, and a phase III trial (PRISM-MEL-301) is now underway to evaluate brenetafusp in combination with nivolumab in treatment-naïve melanoma cases [152].

Furthermore, mRNA vaccines have shown immense promise in designing patient-specific therapies [153]. The mRNA-4157/V940 vaccine is an example of this. The vaccine is engineered to encode up to 34 personalized neoantigens, including PRAME, and is delivered alongside pembrolizumab. Early-phase data from the KEYNOTE-942 trial shows that this combination substantially improved recurrence-free survival in high-risk melanoma patients compared to pembrolizumab alone [153]. Also, the vaccine was well tolerated, which additionally enhances its clinical potential. As a result, it has been granted FDA Breakthrough Therapy Designation and is currently progressing through phase III trials [153].

## 8. Solving Key Barriers in PRAME-Directed Immunotherapy

The dynamic interplay between melanoma cells and the immune system plays a central role in shaping the efficacy of immunotherapeutic approaches. As highlighted in a review on this topic, the process of cancer immunoediting (comprising elimination, equilibrium, and escape) serves as a critical framework for understanding how melanoma evolves under immune pressure and how tumor heterogeneity and immune evasion mechanisms influence treatment response [164]. The integration of omics-based profiling, including transcriptomic and immunogenomic analyses, offers valuable insights into tumor–immune system interactions, revealing potential biomarkers and therapeutic targets that can refine patient stratification and guide the development of combination immunotherapies [164].

**Figure 1:**
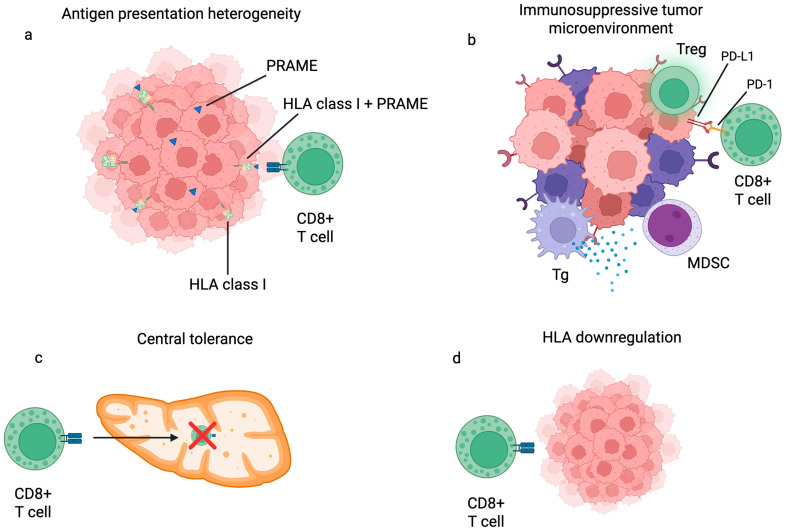
This figure illustrates four major mechanisms by which tumors evade PRAME-targeted immunotherapy. (**a**) This part of the illustration shows how only some tumor cells express PRAME on HLA class I molecules, leading to antigen presentation heterogeneity and incomplete CD8+ T cell recognition [22,36,168]. (**b**) The immunosuppressive tumor microenvironment is depicted. Here, cells like Tregs and MDSCs, along with checkpoint molecules such as PD-L1, inhibit T-cell function [173,174,175,176]. (**c**) This is an illustration of central tolerance, where PRAME-specific T cells are eliminated during thymic development, reducing immune availability [23,38,177]. (**d**) This part of the picture shows reduced HLA class I expression in tumor cells, which impairs antigen presentation and additionally undermines immune detection [178,179,180]. Created in BioRender. Mokos, M. (2025) https://BioRender.com/n8rm2t7, accessed on 5 August 2025. Abbreviations: CD (cluster of differentiation), HLA (human leukocyte antigen), MDSC (myeloid-derived suppressor cell), PRAME (Preferentially Expressed Antigen in Melanoma), PD-1 (programmed cell death protein 1), PD-L1 (programmed death-ligand 1), Tg (tumor-associated macrophage), Treg (regulatory T cells).

One of the primary challenges in advancing PRAME-targeted immunotherapies, including vaccines and ACT, lies in the tumor’s capacity to evade immune detection through multiple mechanisms [38]. Among these, the most critical hurdles are antigen presentation heterogeneity, suppression within the tumor microenvironment (TME), central tolerance via thymic selection, and reduced expression of HLA class I molecules (Figure 1) [38,165,166,167].

A notable complication arises from the inconsistency in tumor antigen presentation. Within a single tumor mass, such as in melanoma, only subsets of cancer cells may display the PRAME antigen on HLA class I molecules, leaving others undetectable to CD8+ T cells [22,36,168]. To tackle this intratumoral variability, researchers are investigating methods to artificially enhance PRAME expression. Since PRAME transcription is epigenetically regulated, specifically by the methylation state of its promoter region, demethylating agents and histone deacetylase (HDAC) inhibitors like DZNep and Decitabine are being explored as adjuvants. These agents can reactivate PRAME expression across tumor cells, potentially making antigen presentation more uniform and improving vaccine and ACT outcomes [39,169,170].

In addition to biological heterogeneity, technical variability in PRAME detection assays also presents a confounding factor in both research and clinical settings. Differences in immunohistochemical protocols, antibody clones, and interpretive thresholds can influence the detection rate and reproducibility of PRAME expression, thereby complicating cross-study comparisons and patient selection for PRAME-targeted therapies [61,171,172]. Establishing standardized assay procedures and diagnostic cutoffs is essential to ensure consistent assessment and optimize clinical utility.

Another formidable barrier to effective immunotherapy is the immunosuppressive nature of the TME. Tumors actively recruit immune-modulatory cells such as regulatory T cells, myeloid-derived suppressor cells (MDSCs), and tumor-associated macrophages. These infiltrating cells release various immunosuppressive factors that blunt CD8+ T-cell responses [173,174,175]. Additionally, malignant cells exploit immune checkpoint pathways by overly expressing molecules such as PD-L1, PD-1, and CTLA-4, which interact with inhibitory receptors on T cells and prevent cytotoxic activity [176]. To fight this inhibitory mechanism, combination therapies involving checkpoint inhibitors (i.e., nivolumab and ipilimumab) alongside PRAME-targeted therapies are under clinical evaluation. Preliminary results from early-phase trials suggest enhanced therapeutic benefits over monotherapy [152].

The elimination of self-reactive T cells during thymic development presents another barrier. Since CTAs such as PRAME are expressed in germline tissues, T cells specific for these antigens are often removed during negative thymic selection to avoid autoimmunity. This immunological censorship limits the pool of available T cells capable of recognizing PRAME-expressing cancer cells, reducing the overall effectiveness of PRAME-based immunotherapies [23,38,177].

Finally, many tumors, including melanoma, avoid immune detection by downregulating HLA class I molecules, thereby impeding CD8+ T-cell recognition [178,179,180]. Therapeutic approaches aiming to restore or enhance HLA-I expression, such as MEK inhibitors and the aforementioned epigenetic modulators, show promise in reversing this evasion tactic [181,182,183,184].

PRAME expression is epigenetically regulated, primarily through DNA methylation of its promoter region. Treatment with DNA methyltransferase inhibitors like decitabine has been shown to upregulate PRAME in multiple tumor cell lines, including melanoma, sarcoma, and leukemia, thus improving antigen visibility to cytotoxic T cells [181]. Additionally, histone deacetylase (HDAC) inhibitors such as entinostat have demonstrated synergistic effects with DNA demethylating agents in enhancing PRAME expression and increasing the immunogenicity of tumor cells [182]. This dual treatment approach has also been linked to enhanced infiltration and activation of CD8+ T cells in preclinical models, potentiating immune-mediated tumor clearance. Furthermore, MEK inhibitors, by targeting oncogenic MAPK signaling, not only restore HLA class I expression but may also sensitize tumors to immune checkpoint blockade and PRAME-directed therapies [183]. A recent study has also revealed that MAPK inhibition can reverse the immune-suppressive effects of tumor-derived extracellular vesicles and reduce myeloid-derived suppressor cell infiltration, further boosting the antitumor immune response [184].

Several ongoing clinical trials are exploring the therapeutic potential of combining PRAME-targeted agents with immune checkpoint inhibitors in melanoma in order to tackle these mechanisms of immune evasion. For instance, the ACTengine^®^ IMA203 trial (Phase I) has demonstrated encouraging efficacy, with a 52.5% overall response rate and a 29% confirmed response rate in PRAME-positive tumors. This study includes combination arms with nivolumab, suggesting potential synergy between PRAME-specific TCR therapies and PD-1 blockade [149]. Another innovative approach is being evaluated in a Phase I trial of IMC-F106C, a bispecific ImmTAC molecule targeting both PRAME and CD3. This agent showed a 58% disease control rate and manageable toxicity in heavily pre-treated cutaneous melanoma patients, supporting its further development in combination regimens [151]. Building upon these early findings, the ongoing Phase III PRISM-MEL-301 trial is comparing IMC-F106C plus nivolumab versus standard nivolumab regimens in previously untreated HLA-A*02:01+ advanced melanoma patients, aiming to improve progression-free and overall survival outcomes [152].

## 9. Conclusions

PRAME has emerged as a diagnostically and therapeutically relevant biomarker in melanoma. Its limited expression in normal tissues and consistent overexpression in malignant melanocytic lesions are proof of its high specificity. This is the reason why PRAME is such a valuable tool in distinguishing melanoma from benign nevi, particularly in diagnostically challenging cases such as acral, mucosal, and spitzoid lesions.

When it comes to the future perspectives of PRAME, its integration into routine clinical practice is expected to evolve significantly. Since immunotherapy continues to develop, it is going to be crucial to refine PRAME-targeting strategies (such as TCR-engineered T cells, bispecific ImmTACs, and personalized mRNA vaccines). Furthermore, expanding our understanding of PRAME’s regulation could be essential for the development of synergistic combination therapies that enhance antigen presentation and immune responsiveness. To ensure consistency and clinical reliability, multicenter studies are needed to standardize PRAME immunohistochemistry interpretation across laboratories and patient populations. Large clinical trials and standardized diagnostic protocols will be of huge importance in evaluating PRAME’s role across diverse melanoma subtypes and stages. Finally, PRAME is not only a promising biomarker, but also as a cornerstone of precise immuno-oncology.

In summary, PRAME is an example of the convergence of diagnostic precision and therapeutic innovation in melanoma. Ongoing translational research will be essential to optimize its clinical utility and to integrate PRAME-directed approaches into personalized melanoma care.

## Figures and Tables

**Table 2:** Comparison of the diagnostic utility of PRAME and other melanocytic markers.

Marker	Specificity	Sensitivity	Additional Notes	Reference Numbers
S100	Low	High	Stains both benign and malignant melanocytes	[4,65,66,67,68,69]
SOX10	Low	High	Limited specificity similar to S100	[4,65,66,67,68,69]
Melan-A	Low	High	Difficulties in differentiating benign from malignant lesions	[70]
HMB-45	Moderate	Moderate	Slightly improved specificity compared to others	[67,71]
PD-L1	Variable	Variable	Predictive value inconsistent; depends on tumor microenvironment	[72,73,74,75]
PRAME	High (86.4% negative in benign nevi)	92% in primary and metastatic melanoma; 85.4% in metastases	Higher specificity than traditional markers	[22,66,76,77,78,79]

**Table 3:** Clinical trials investigating PRAME-targeted immunotherapies in melanoma and other solid tumors.

Trial Name	Main Observations	Clinical Phase	Reference Number
Safety & Activity of Controllable PRAME-TCR Therapy in Previously Treated AML/MDS or Metastatic Uveal Melanoma	TCR-engineered autologous T-cell therapy targeting PRAME in metastatic uveal melanoma; features a rimiducid-controlled safety switch.	Phase II	[148]
ACTengine^®^ IMA203/IMA203CD8 as Monotherapy or in Combination With Nivolumab in Recurrent and/or Refractory Solid Tumors (ACTengine)	Phase I trial showed a manageable safety profile, 52.5% overall response rate, 29% confirmed response in PRAME-positive tumors; supports further development.	Phase I	[149]
Safety and Immune Response to a Multi-component Immune Based Therapy (MKC1106-PP) for Patients With Advanced Cancer.	Triggered CD8+ T-cell expansion in 15 of 24 prostate cancer patients; some showed disease stability over 6 months (this included one out of ten patients with metastatic melanoma).	Phase I	[150]
Safety and Efficacy of IMC-F106C as a Single Agent and in Combination With Checkpoint Inhibitors	Bispecific ImmTAC molecule targeting PRAME and CD3; showed 58% disease control rate and median PFS of 4.2 months in heavily pre-treated cutaneous melanoma; 42% of PRAME-positive patients showed molecular responses via ctDNA; adverse events were manageable (e.g., mild cytokine release syndrome, rash)	Phase I	[151]
IMC-F106C Regimen Versus Nivolumab Regimens in Previously Untreated Advanced Melanoma (PRISM-MEL-301) (PRISM-MEL-301)	Phase III randomized global study evaluating IMC-F106C (PRAME × CD3) in combination with nivolumab in previously untreated HLA-A*02:01+ advanced melanoma patients. Aims to improve PFS, OS, and response rates versus standard nivolumab regimens. Trial includes patients with cutaneous and select non-cutaneous melanoma subtypes. No results published yet.	Phase III	[152]
An Efficacy Study of Adjuvant Treatment With the Personalized Cancer Vaccine mRNA-4157 and Pembrolizumab in Participants With High-Risk Melanoma (KEYNOTE-942)	Improved recurrence-free survival in high-risk melanoma patients; well tolerated; received FDA Breakthrough Therapy Designation.	Phase IIb	[153]
